# Multidomain Biomarkers as Predictors of Cardiovascular Risk in Acute Coronary Syndrome: A Prospective Evaluation

**DOI:** 10.3390/ijms27052476

**Published:** 2026-03-07

**Authors:** Guadalupe Estela Gavilánez-Chávez, Maria G. Zavala-Cerna, Sandra Guzmán-Silahua, Luz Rebeca Rodríguez-Rivera, Cristo F. Urzua-Ortega, Ernesto Germán Cardona-Muñoz, Eduardo Chuquiure-Valenzuela, Benjamín Rubio-Jurado, Arnulfo Hernán Nava-Zavala

**Affiliations:** 1 Servicio de Urgencias, Hospital General Regional 46, Instituto Mexicano del Seguro Social, Guadalajara 44910, Mexico; dragavilanez@gmail.com (G.E.G.-C.);; 2 Laboratorio de Investigación en Inmunología, Decanato Medicina, Universidad Autónoma de Guadalajara, Zapopan 45129, Mexico; maria.cerna@edu.uag.mx (M.G.Z.-C.); cristo.urzua@edu.uag.mx (C.F.U.-O.); 3 Unidad de Investigación Epidemiológica y en Servicios de Salud, Centro Medico Nacional de Occidente, Órgano de Operación Administrativa DesconcentradaJalisco, Instituto Mexicano del Seguro Social, Guadalajara 44340, Mexico; 4 Programa Internacional de Medicina, Universidad Autónoma de Guadalajara, Zapopan 45129, Mexico; 5 Programa de Doctorado en Farmacología, Departamento de Fisiología, Centro Universitario Ciencias de la Salud, Universidad de Guadalajara, Guadalajara 44340, Mexico; 6 Instituto Nacional de Cardiológia “Ignacio Chavez”, Ciudad de Mexico 14080, Mexico; 7 Departamento Clínico de Hematología, División Onco-Hematologia, Unidad Medica de Alta Especialidad, Hospital de Especialidades, Centro Médico Nacional de Occidente, Instituto Mexicano del Seguro Social, Guadalajara 44340, Mexico; 8 Departamento de Inmunología y Reumatología del Hospital General de Occidente, Secretaría de Salud Jalisco, Guadalajara 45170, Mexico

**Keywords:** acute coronary syndrome, biomarkers, MACE, autoimmunity

## Abstract

Acute coronary syndrome (ACS), driven by inflammation and thrombosis, remains a leading cause of morbidity globally. While traditional risk scores are useful, the prognostic value of combining inflammatory and autoimmune biomarkers remains understudied. This study aimed to evaluate the predictive role of high-sensitivity C-reactive protein (hs-CRP), platelet factor 4 (PF4), D-dimer, and antiphospholipid antibodies (anticardiolipin and anti-β2-glycoprotein I) for the development of major adverse cardiovascular events (MACE) in patients with ACS. We conducted a prospective cohort study at a tertiary referral center in Mexico. A total of 103 patients admitted with confirmed ACS were included. Blood samples were collected upon admission to measure biomarker levels. Participants were followed for 30 days. The primary outcome was the occurrence of MACE, defined as reinfarction, death, percutaneous coronary intervention, or bypass surgery. Multivariate logistic regression analysis was performed to identify independent predictors, adjusting for age, smoking, and comorbidities. MACE occurred in 51.4% of participants. Patients with adverse outcomes were significantly older and had longer hospital stays (*p* < 0.05). In the biomarker analysis, PF4 and hs-CRP demonstrated high sensitivity (98%) but low specificity. In the multivariate analysis, IgG anti-β2-glycoprotein I (*p* < 0.001) and D-dimer (*p* = 0.024) emerged as significant independent predictors of MACE. Conversely, IgM isotypes did not show independent predictive value. Beyond traditional risk factors, markers of coagulation (D-dimer) and autoimmunity (IgG anti-β2-glycoprotein I) are independent predictors of short-term adverse events in ACS patients. Integrating these multidomain biomarkers into clinical assessment may enhance risk stratification and prognostic accuracy.

## 1. Introduction

Acute coronary syndrome (ACS) is the leading cause of morbidity and mortality worldwide [1], and its primary cause is the erosion or rupture of an atheromatous plaque; ACS includes unstable angina (UA), acute ST-elevation myocardial infarction (STEMI), or non-ST-segment elevation myocardial infarction (NSTEMI) [2]. The outcome depends on the extension and duration of the obstructed vessel, the existence of collateral circulation, and/or the presence of vasospasm at the time of plaque rupture [3]. Atherosclerosis usually precedes the rupture of an atheromatous plaque, with coronary inflammation being a primary stimulus [4]. In its initial phase, the inflammatory response promotes platelet activation and coagulation [5], described as a promoter for thrombotic events [6]. Both inflammation and platelet activation play central roles in the pathophysiology and prognosis of ACS. Numerous studies have investigated the prognostic value of C-reactive protein, an inflammatory biomarker, other composite indices (neutrophil-to-lymphocyte ratio, systemic immune–inflammation index), and platelet activation factors such as platelet factor 4, mean platelet volume, and beta-thromboglobulin in the prediction of adverse outcomes in ACS patients. Evidence consistently demonstrates their role in inflammatory and platelet activation, making them independent predictors for short- and long-term adverse cardiovascular events, including mortality, major adverse cardiovascular events (MACE), and recurrent ischemic events [7,8,9,10,11].

Apart from platelet activation, the coagulation system has also been involved in ACS development [12,13]; importantly, increased D-dimer concentration in the plasma reflects an ongoing thrombosis and fibrinolysis, correlating with plaque rupture and thrombus burden [14]; multiple large cohort studies have consistently shown that elevated D-dimer levels are independently associated with higher risks of all-cause death, cardiac death, and major adverse cardiovascular events (MACE). These adverse events include heart failure (HF), ST-segment elevation myocardial infarction (STEMI), non–ST-segment elevation myocardial infarction (NSTEMI), unstable angina, and death in ACS patients [15,16,17,18]. Meta-analyses report that patients with high D-dimer levels have a 1.2–2.4 times higher risk of adverse events or mortality compared to those with low levels [19,20].

In patients with autoimmune diseases, thrombosis in the coronary vasculature is rare; however, the positivity for anticardiolipin has been associated with increased risk for acute myocardial infarction in young patients (<40 years of age) [21]. Nevertheless, there is no clear link to mortality or reinfarction in ACS [22]. Anti-beta 2 glycoprotein I antibodies (anti-β2GP1) have been described with higher specificity for thrombosis, hence the great interest in using them as biomarkers for thrombosis development [23]. These antibodies are known to promote activation of endothelial cells, monocytes, and neutrophils [24]; however, their predictive role in cardiovascular outcomes [25], and coronary lesions remains unknown [26,27].

Currently, dozens of molecules are known to be involved in the atherosclerotic process [28], and in some cases, their use as prognostic risk markers for cardiovascular outcomes has been clearly identified; however, consistency has not been achieved for autoimmunity biomarkers and therefore, their predictive value seems compromised [29]. In general, the role of autoimmunity markers for ACS adverse events is less clear. The integration of these biomarkers with established clinical risk and inflammatory biomarkers further enhances risk stratification and outcome prediction in ACS. For this purpose, we undertook a prospective study to identify the predictive role of hs-CRP, platelet factor 4 (PF4), anticardiolipin (IgM), and anti-β2GP1 antibodies for the development of MACE in patients with acute coronary syndrome after a 30-day follow-up period. We expect that this information can be used by clinicians to create a broader biomarker analysis and prevent severe outcomes in patients with ACS.

## 2. Results

**Figure 1:**
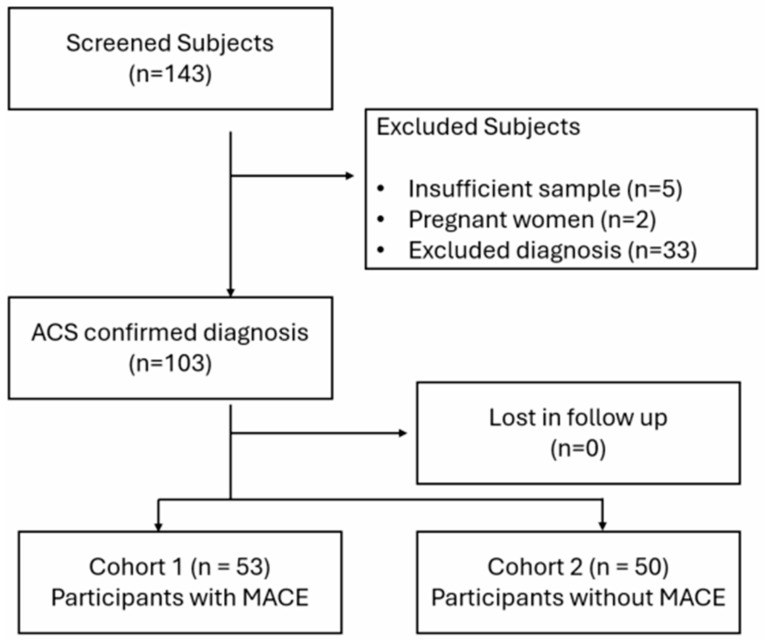
Study diagram.

A total of 143 participants admitted to the ED with a diagnosis of ACS were screened for eligibility for the study, of which 103 participants met the inclusion criteria. Reasons for exclusion can be found in the study diagram (Figure 1). Given the 30-day follow-up period, we experienced no losses during this short period.

The participants’ average age was 64.62 ± 14.17. Of the participants, 69 (67.1%) were men, and 34 (33.0%) were women. We identified 53 (51.45%) patients with MACE outcomes, including 5 (9%) with unstable angina, 21 (40%) with ST-segment elevation myocardial infarction (STEMI), 18 (34%) with non-ST-segment elevation myocardial infarction (NSTEMI), and 9 (17%) deaths, as well as 50 (48.54%) without MACE outcomes; overall patient mortality was 9.7%.

We then analyzed demographics, clinical variables, and the presence of comorbidities in association with the presence of MACE, and we found smoking, increased age, and in-hospital stay (IHS) to be significantly associated with the presence of MACE (Table 1). We then performed an analysis to detect significant changes in means of inflammation, platelet activation, and autoimmunity biomarkers associated with the presence of MACE (Table 2). Significant associations were found for D-dimer, hs-CRP, and PF4. Although *p* values were significant, they did not reach the level of significance obtained by troponin I.

Afterwards, we conducted an analysis to test biomarker sensitivity, specificity, and predictive values for MACE in comparison with those for troponin I, where the highest sensitivity values were found for PF-4 and Hs-CRP (98%), with even higher values compared to troponin I; D-dimer levels were slightly below this threshold, and antibodies displayed a sensitivity of around 40%.

With respect to specificity, as expected, the highest values were for CPK-MB (82%) and troponin I (86%); interestingly, in comparison to inflammatory and coagulation biomarkers, autoimmunity biomarkers performed better, with 79% for anticardiolipin and 78% for anti β2-glycoprotein-I (Table 3). For PF4, specificity was low and even lower for hs-CRP, with 44% and 20%, respectively. Positive (PPV) and negative predictive value (NPV) performed similarly only for D-dimer in comparison to troponin I; hs-CRP yielded higher NPV compared to the standard (91 vs. 82).

**Figure 2:**
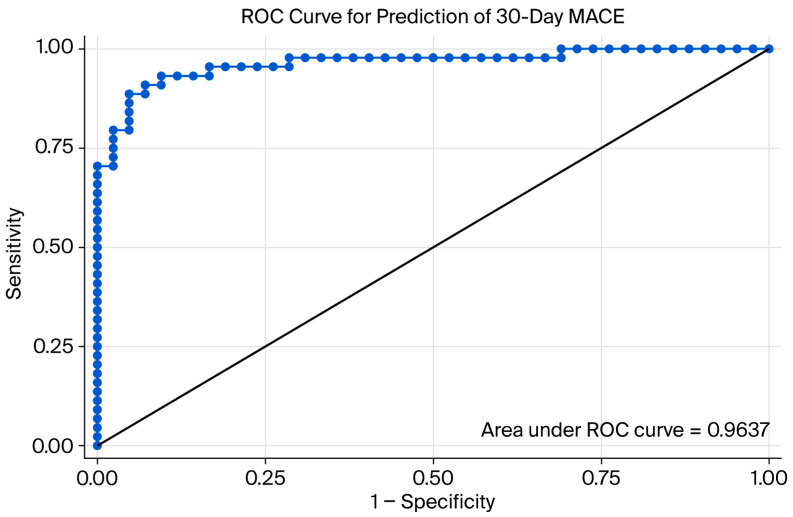
Receiver operating characteristic (ROC) curve for the multivariable logistic regression model predicting 30-day major adverse cardiovascular events (MACE) in patients with acute coronary syndrome. The model included hs-CRP, platelet factor 4, anticardiolipin IgM, anti-β2 glycoprotein I antibodies, age, smoking status, days of intra-hospital stay, and comorbidities. The area under the curve (AUC) was 0.9637, reflecting the model’s ability to distinguish between patients with and without MACE.

Finally, we undertook single and multivariate analysis to report on the contribution of each biomarker to the presence of MACE; in the multivariate analysis, we adjusted for age, sex, smoking, in-hospital stay, and the presence of comorbidities which are known to be confounding effects for MACE development (Table 4). We tested the multivariable model for the prediction of MACE, which is represented in Figure 2.

## 3. Discussion

This prospective study aimed at evaluating the predictive value of a multidomain biomarker panel involving inflammation, coagulation, and autoimmunity in patients with acute coronary syndrome (ACS). Our main finding indicates that beyond traditional biomarkers like troponin I and hs-CRP, autoantibodies—specifically IgG anti-β2-glycoprotein I (anti-β2-glycoprotein-I) and anticardiolipin antibodies (aCL)—along with D-dimer, emerged as independent predictors for MACE at the 30-day follow-up.

In our cohort, 51.4% developed at least one MACE, with a mortality rate of 9.7%. This incidence is notably higher compared to the results of some reports in the literature, which describe MACE rates as low as 9.9% in similar follow-up periods [30]. However, our findings align with studies involving high-risk populations, such as those with chronic kidney disease, where MACE rates reach up to 40.2% [31]. The elevated incidence in our study can be attributed to the complex clinical profile of our patients, characterized by advanced age (64.6 years) and a high prevalence of comorbidities, particularly hypertension and diabetes (73.8%). Specifically, the prevalence of systemic arterial hypertension in our cohort far exceeded values reported in comparable studies, which range from 47.8% [32] to 52.7% [33]; male sex has also been previously associated to this outcome [34]; however, in our cohort, sex was not associated to MACE.

In myocardial necrosis, the biomarker superiority of troponin was confirmed in our study [32,35]. High-sensitivity cardiac troponin detection tests have become the gold standard for diagnosis, due to their ability to detect of troponins at a much lower concentration than those in classical assays. With high sensitivity and specificity, they allow for fast recognition of even small infarcts [36]. Therefore, troponins play a crucial role in the diagnostic algorithm of the latest universal definition of infarction [37]. Furthermore, they can be robust long-term prognostic markers of adverse events and mortality [38].

In our study, troponin I solidified its position as the biomarker with the highest overall discriminative capacity. Consistent with our findings, patients who developed MACE exhibited significantly higher admission concentrations compared to those who did not (*p* = 0.001). Crucially, regarding diagnostic performance, while other biomarkers in our panel (such as PF4 or hs-CRP) achieved superior individual sensitivity values, troponin I demonstrated the best global performance for predicting MACE in 30 days. This finding reaffirms its dual utility: it serves not only as the gold standard for the diagnosis of myocardial necrosis but also as a robust early prognostic marker for adverse outcomes in ACS. These results align with major risk scores like HEART and GRACE, where troponin levels drive risk stratification [39].

Additionally, we found that D-dimer, hs-C-reactive protein, platelet factor-4, anticardiolipin antibodies, and anti-beta-2 glycoprotein antibodies were significantly associated in a model to predict the development of MACE in the 30-day follow-up of our study, demonstrating their relevant use for patients upon admission to the emergency department.

In our study, high-sensitivity C-reactive protein (hs-CRP) demonstrated a diagnostic sensitivity of 98%, consolidating its role as a highly sensitive systemic marker in the context of acute coronary syndrome (ACS). We observed significantly higher median levels in patients who developed MACE (1502 pg/mL) compared to those who did not (825 pg/mL), reinforcing the premise that vascular inflammation is a critical determinant of short-term outcomes.

Biologically, hs-CRP serves as a broad indicator of inflammation and an acute-phase reactant stimulated by IL-6, which subsequently drives the synthesis of cytokines promoting leukocyte migration toward damaged vascular tissue [14]. Given its circulating half-life of approximately 19 h and its function in opsonizing endogenous and exogenous substances for clearance [40], the elevated levels observed in our cohort reflect a persistent and active inflammatory state. This inflammatory response and the associated endothelial lesions are critical events that, over time, lead to the establishment and instability of atherosclerotic plaque, subsequently serving as a nidus for platelet aggregation and thrombosis [41]. The high-sensitive CRP (hs-CPR) assay has enabled the precise measurement of this biomarker as a tool to improve risk stratification [5].

Our findings regarding the prognostic value of this biomarker align closely with the large-scale cohort, which established a relationship between hs-CRP levels and 30-day mortality, reporting an adjusted hazard ratio (aHR) of 1.66 (95% CI 1.43–1.92) for patients with mildly elevated levels (10–15 mg/L) and an even higher risk (aHR 2.00) for those exceeding 15 mg/L, independent of troponin status [42]. This statistical evidence directly aligns with our 30-day follow-up results, confirming that systemic inflammation represents a distinct pathophysiological axis offering prognostic value for immediate mortality and adverse events beyond myocardial necrosis markers alone. Furthermore, the clinical utility of a baseline assessment for short-term risk is supported by a trial analysis, which found that elevated baseline hs-CRP levels were significantly associated with MACE (OR 1.36 per standard deviation increase, *p* < 0.001), specifically during the acute and subacute follow-up phases (16 weeks), mirroring the early event divergence observed in our 30-day window [43]. This is further reinforced by the meta-analysis, which confirmed that baseline hs-CRP is a universal predictor of adverse clinical outcomes in coronary artery disease, with pooled data indicating a significantly elevated relative risk (RR) for short-term cardiovascular events in the highest CRP quartiles [44].

The cytokine platelet factor 4 (PF4) is a biomarker for platelet activation; its function is to maintain hemostasia after inflammatory responses in the body [45]. We observed higher concentrations of PF4 in patients with MACE. Although this difference did not reach statistical significance (*p* = 0.064), PF4 demonstrated a sensitivity of 98%. This suggests its potential utility as a screening tool to identify patients with early risk of thrombotic complications, reflecting the central role of platelet activation in the acute phase of atherothrombosis [46].

These results are widely consistent with a recent systematic review and meta-analysis which reaffirmed that patients with ACS exhibit significantly higher PF4 levels than do healthy controls, with a standardized mean difference (SMD) reaching 2.01 in acute cases versus 0.96 in stable disease [47].

In our study, autoimmunity biomarkers assessed independently did not present with a high predictive value, which could be related to the fact that these pathways converge at different times and could participate through other mechanisms; however, more studies are required to corroborate these data.

The search for mechanisms explaining residual cardiovascular risk has highlighted the immunological component. Our findings support the hypothesis that autoantibodies actively contribute to the development of a prothrombotic state and subsequent MACE. Among the most widely studied are antiphospholipid antibodies (aPL), specifically anticardiolipin (aCL), and anti-β2-glycoprotein I (anti-β2-glycoprotein-I), whose thrombogenic mechanisms are well-documented.

This pathophysiological process begins with the binding of antiphospholipid antibodies to the β2-glycoprotein I complex on the surface of endothelial cells. This interaction triggers endothelial activation, converting the surface from anti-thrombotic to pro-thrombotic. This shift induces the expression and secretion of tissue factor—the primary initiator of the extrinsic coagulation pathway—along with increased adhesion molecules and Von Willebrand factor, promoting platelet aggregation. Furthermore, these autoantibodies may exacerbate atherosclerosis by promoting lipoprotein uptake by macrophages. The formation of immune complexes involving LDL and β2GP1 facilitates the conversion of macrophages into foam cells, leading to the release of inflammatory mediators that contribute to atherosclerotic plaque instability. These mechanisms collectively establish a chronic state of inflammation and thrombosis, directly precipitating MACE [48].

In the general healthy population, the prevalence of antiphospholipid antibodies is low, from 1% to 5% [49]. In contrast, patients with established autoimmune diseases exhibit much higher rates; for instance, antiphospholipid antibodies are present in up to 20–40% of patients with systemic lupus erythematosus (SLE) [49], and its presence is considered a classification criterion for antiphospholipid syndrome (APS) [50].

Interestingly, our study identified a significant association between these antibodies and MACE in an unselected ACS cohort. This suggests that even in the absence of a formal diagnosis of an autoimmune disease, the prevalence of these autoantibodies—likely acting through the pro-thrombotic and pro-inflammatory mechanisms described above—plays a critical role in the prognosis of patients with acute coronary syndrome, mirroring the increased cardiovascular risk observed in classic autoimmune conditions.

A pivotal finding of our study is the independent predictive value of antiphospholipid antibodies (aPL). Our multivariate analysis showed that IgG anti-β2GP1 (*p* < 0.001) and IgM anticardiolipin (*p* = 0.044) were independently associated with MACE in this unselected ACS population.

Our findings are strongly supported by existing literature. For instance, a meta-analysis demonstrated that elevated IgM anti-cardiolipin titers are independently associated with an increased risk of atherosclerotic cardiovascular disease (OR 1.27; 95% CI 1.08–1.30) [48]. Furthermore, our results regarding the prognostic impact of these antibodies mirror previous investigations in which ACS patients with positive antiphospholipid antibodies had a significantly higher risk for adverse events [51]. This consistency across studies reinforces the hypothesis that autoimmunity contributes to a residual inflammatory risk that traditional therapies may not fully address.

Our study exhibits important limitations; first, it is a single-center study with a relatively small sample size (*n* = 103), which may limit the generalizability of the results. Our use of single and multiple variable analysis yields a limited sample size and number of events observed, so a subanalyses each individual MACE component was not conducted; instead, we used the MACE analysis strategy as a cumulated outcome. Future studies with a larger sample size, including variables with the predictive potential found in this study and possibly additional variables, are required to accurately elucidate the correlations and predictability. Second, we measured biomarkers at a single time point (admission); serial measurements could provide a better insight into the kinetics of these markers. Finally, we did not perform long-term follow-up beyond 30 days. Despite these limitations, our findings suggest the hypothesis of immune-mediated thrombosis in ACS.

## 4. Materials and Methods

We undertook a prospective cohort study in compliance with the ethical standards established in the Declaration of Helsinki of 1964 and subsequent amendments [52] and the study was approved by the Health Research and Ethics Committee No. 1306 of IMSS in Guadalajara, Jalisco, Mexico, with the number R-2018-1306-013.

### 4.1. Study Participants

Participants of this study were those who presented at the emergency department of a tertiary referral hospital within the social security system in Mexico (IMSS). Participants were screened for the following inclusion criteria: >18 years of age, admitted for ACS, availability of blood samples at the time of admission, IMSS beneficiaries, and who agreed to participate in the study by signature of informed consent. Participants were excluded if they had insufficient blood samples, were pregnant, were in a post-operative period of less than 72 h, polytraumatized or transplanted, we also excluded participants with an established diagnosis of substance use or chronic renal failure. Initial diagnosis was confirmed by measurement of troponin I, and this test was used as a reference for the performance of additional biomarkers; participants were followed for a 30-day period to register the presence of cardiovascular outcomes.

### 4.2. Blood Samples

Blood samples were recovered from the remaining blood samples collected upon arrival at the ED, serum and plasma were separated and aliquoted into a 2 mL micro-vial, identified with a folio number and patient’s initials, and stored at −80 °C until tested for PF4, d-dimer, hs-CRP, anticardiolipin antibodies, and anti-β2GP1.

### 4.3. Study Outcomes

We used the term MACE for major adverse cardiovascular events, which include acute myocardial infarction with or without ST-segment elevation, percutaneous coronary intervention, bypass surgery, reinfarction, or death.

After 30 days of follow-up, we categorized patients as With MACE, if they exhibited at least one major adverse cardiovascular event, or Without MACE, if they did not exhibit any event.

### 4.4. Laboratory Determinations

For FP4 measurement, the human CXCL4/PF4 Elisa kit (R&D Systems, Inc., Minneapolis, MN, USA) was used, with minimum and maximum detection values of 20.5 and 15,000 pg/mL, respectively. The cut-off value was 1667 pg/mL.

For hs-CRP, we used a MYBIOSOURCE sandwich-type Elisa CPR HS kit (quantitative, MyBioSource, Inc., San Diego, CA, USA); the minimum and maximum detection values were 125 pg/mL and 1000 pg/mL, respectively. The cut-off value was 1000 pg/mL.

D-dimer was evaluated with the VIDAS D-DIMER exclusion II kit (DEX 2, bioMérieux S.A., Marcy l’Étoile, France). The minimum and maximum detection values were 45 and 10,000 ng/mL, respectively. The cut-off was less than 500 ng/mL.

For the measurement of IgG and IgM anti-β2GPI antibodies, the Elisa MYBIOSOURCE kit (MyBioSource, Inc., San Diego, CA, USA) was used, with a detection range of 0.781–50 U/mL and a cut-off value of 12.5–20 U/mL.

Finally, anticardiolipin IgM was measured with a MYBIOSOURCE human anticardiolipin IgM Elisa Sandwich-type booklet kit (MyBioSource, Inc., San Diego, CA, USA), with a minimum detection of 3–48 U/mL, and a cut-off value of 20 U/mL.

We performed all tests according to each manufacturer’s instructions, and afterwards, spectrophotometry at 450 nm was performed for each 96-well plate; the absorbance values were obtained with a MultiSan Go Thermo Scientific^®^ (Waltham, MA, USA), and the results were analyzed and transformed using Skanlt™ software 6.1.1.

### 4.5. Statistical Analysis

For quantitative variables, means, standard deviation, medians, and ranges were used; for qualitative variables, proportions and percentages were used. In the inferential phase, the quantitative variables were compared with the use of a Student’s *t* or Mann–Whitney U test, and the Chi-square or Fisher’s exact test were used for the qualitative variables. We also calculated values for sensitivity, specificity, positive predictive value, and negative predictive value to establish biomarkers for possible use. The statistical analysis was carried out using the SPSS V 24 program; a *p*-value ≤ 0.05 was considered significant.

## 5. Conclusions

In conclusion, our study presents evidence that supports a multidomain set of biomarkers that accurately predict the development of MACE in ACS patients; the use of these biomarkers alone is specifically restricted to autoimmunity biomarkers, but in conjunction with clinical data, inflammatory biomarkers, and others related to coagulation, their usefulness can be suggested, although larger cohort studies will contribute to replicate and better define their role as predictors for risk stratification in ACS patients.

## Figures and Tables

**Table 1:** Demographic and clinical variables in ACS patients.

Variable	With MACE *n* = 53	Without MACE *n* = 50	*p*	RR	95% CI
Age (years), $\bar{X}$ ± SD	68.3 ± 12.25	61.0 ± 15.76			
Elderly category					
>65 years, *n* = 61	35/61 (57%)	26/61 (43%)	**0.009** ^a^	**1.56**	**1.94–2.61**
<65 years, *n* = 42	18/42 (43%)	24/42 (57%)			
Gender, *n* (%)					
Men, *n* = 70	37/70 (53%)	33/70 (47%)	0.674 ^b^	1.1	1.07–1.70
Women, *n* = 33	16/33 (48%)	17/33 (52%)			
Smoking, *n* (%)	25 (47.1)	11 (22.0)	**0.008** ^a^	**2.9**	**2.10–3.30**
Evolution at admission (h), $\bar{X}$ ± SD	12.2 ± 10.5	13.6 ± 11.5	0.713 ^a^	1.05	0.95–1.95
IHS, d, Me, Vmin–Vmax	7.5 (1–72)	3.0 (1–18)	**0.002** ^a^	1.62	1.14–2.29
Comorbidities, *n* (%)					
SAH	38 (71.6)	38 (76.0)	0.503 ^b^	1.18	0.80–1.70
dyslipidemia	21 (39.6)	16 (32.0)	0.543 ^b^	1.10	0.49–1.40
diabetes mellitus 2	28 (52.8)	28 (56.1)	0.844 ^b^	1.06	0.75–1.50
previous ACS	38 (71.6)	2 (4.0)	**0.001** ^b^	2.80	1.96–4.56
previous thrombosis	14 (26.4)	19 (38.1)	0.206 ^b^	1.52	1.48–2.65
rheumatoid arthritis	1 (1.8)	2 (4.0)	0.604 ^b^	2.22	1.2–23.00
COPD	6 (11.3)	1 (2.0)	0.274 ^b^	0.36	0.80–1.70

$\bar{X}$: mean, SD: standard deviation, %: percentage, h: hours, d: days, HIS: in-hospital stay, Me: median, Vmin–Vmax: minimum value–maximum value, SAH: systemic arterial hypertension. Previous thrombosis includes pulmonary thromboembolism, ischemic cerebrovascular events, and deep vein thrombosis of the limbs. COPD: chronic obstructive pulmonary disease. Calculated with ^a^ Student’s *t*-test, ^b^ Chi-square. Bold values indicate statistical significance with a *p*-value less than or equal to 0.05.

**Table 2:** Comparison of biomarkers in 103 ACS patients according to the occurrence of MACE.

Biomarker	With MACE *n* = 53	Without MACE *n* = 50	*p*
Troponin I ng/L *	328 (7.3–40,000)	8.1 (1.5–21,612)	**0.001**
D-dimer ng/mL *	574 (95–20,401)	214 (72–7200)	**0.048**
IgM anti-cardiolipin U/mL *	11.1 (6.4–50.9)	10.2 (4.3–45.5)	0.434
IgM anti β2-glycoprotein-I U/mL *	8.5 (0.14–52)	5.27 (5.2–55.5)	0.312
IgG anti β2-glycoprotein-I U/mL *	39.2 (0.04–42.3)	0.57 (0.04–42.1)	**0.001**
Hs-CRP pg/mL *	1502 (125–5018)	825 (10.5–2830)	**0.050**
Platelet factor 4 pg/mL *	15,105 (8018–15,900)	10,905 (418–14,800)	**0.064**

ACS: acute coronary syndrome, HS-CRP: high sensitivity c-reactive protein, IgM: immunoglobulin M, ng/L: nanograms per liter, U/mL: units per milliliter, ng/mL: nanograms per milliliter, pg/mL: picograms per milliliter, *: I (range). Calculated using Mann–Whitney U. Bold values indicate statistical significance with a *p*-value less than or equal to 0.05.

**Table 3:** Biomarker values of sensitivity, specificity, and predictive values for MACE in ACS patients.

Biomarker	Sensitivity %	Specificity %	PPV %	NPV %
Platelet factor 4	98	44	52	66
Hs-CRP	98	20	56	91
D-dimer	79	75	75	80
IgM anticardiolipin	43	79	46	67
IgM anti β2-glycoprotein-I	41	78	66	55
IgG anti β2-glycoprotein-I	88	89	87	70
Troponin I	86	82	86	82
CPK-MB	49	86	78	61

Hs-CRP: high sensitivity C-reactive protein, IgM: immunoglobulin M, CPK-MB: creatine phosphokinase mb fraction.

**Table 4:** Associations between MACE and risk factors in ACS patients.

Variable	Single Variable			Model 1 (Multivariate)		
	OR	CI	*p*	OR	CI	*p*
Troponin I	1.00	1.00–1.00	**0.025**	1.00	1.00–1.00	**0.048**
D-dimer	1.00	1.00–1.00	**0.042**	1.00	0.99–1.00	**0.024**
IgM anti-cardiolipin	1.01	0.96–1.06	0.689	1.21	0.99–1.25	**0.044**
IgM anti β2-glycoprotein-I	0.98	0.96–1.01	0.209	0.95	0.91–1.03	0.347
IgG anti β2-glycoprotein-I	1.08	1.05–1.11	<**0.001**	1.13	1.05–1.21	<**0.001**
Hs-CRP	1.00	1.00–1.00	**0.045**	1.00	0.99–1.00	**0.028**
Platelet factor 4	1.00	0.99–1.00	0.291	0.99	0.99–1.00	0.659
Age				1.10	1.00–1.12	**0.030**
Smoking				5.68	1.02–16.8	0.076
IHS				1.32	1.04–1.69	**0.021**
Comorbidities				1.70	0.04–77.19	0.786

Model 1: adjusted for age (years), smoking, IHS (in-hospital stay), and the presence of comorbidities (R^2^ = 0.6461, *p* < 0.001). Bold values indicate statistical significance with a *p*-value less than or equal to 0.05.

## Data Availability

The original contributions presented in this study are included in the article. Further inquiries can be directed to the corresponding authors.

## Abbreviations

ACS	Acute coronary syndrome
aCL	Anticardiolipin antibodies
anti-β2GP1	Anti-beta-2 glycoprotein I antibodies
aPL	Antiphospholipid antibodies
hs-CRP	High-sensitivity C-reactive protein
MACE	Major adverse cardiovascular events
PF4	Platelet factor 4

## References

[1] Odeberg J., Halling A., Ringborn M., Freitag M., Persson M.L., Vaara I., Rastam L., Odeberg H., Lindblad U. (2025). Markers of inflammation predicts long-term mortality in patients with acute coronary syndrome—A cohort study. BMC Cardiovasc. Disord..

[2] Michailovich Chaulin A. (2023). Diagnostic Role and Methods of Detection of Cardiac Troponins: An Opinion from Historical and Current Points of View. Curr. Cardiol. Rev..

[3] White H., Thygesen K., Alpert J.S., Jaffe A. (2014). Universal MI definition update for cardiovascular disease. Curr. Cardiol. Rep..

[4] Vidula H., Tian L., Liu K., Criqui M.H., Ferrucci L., Pearce W.H., Greenland P., Green D., Tan J., Garside D.B. (2008). Biomarkers of inflammation and thrombosis as predictors of near-term mortality in patients with peripheral arterial disease: A cohort study. Ann. Intern. Med..

[5] Libby P. (2012). Inflammation in atherosclerosis. Arterioscler. Thromb. Vasc. Biol..

[6] Hackam D.G., Spence J.D. (2019). Antiplatelet Therapy in Ischemic Stroke and Transient Ischemic Attack. Stroke.

[7] Bao Q., Liu T., Song H., Bao W., Fan W. (2025). Prognostic Role of Inflammatory Hematologic Indices in Predicting Acute Coronary Syndrome in Elderly Patients with Chronic Coronary Syndrome. J. Inflamm. Res..

[8] Li Q., Ma X., Shao Q., Yang Z., Wang Y., Gao F., Zhou Y., Yang L., Wang Z. (2022). Prognostic Impact of Multiple Lymphocyte-Based Inflammatory Indices in Acute Coronary Syndrome Patients. Front. Cardiovasc. Med..

[9] Cao F., Jiang J.-J., Zhang G., Liu J., Xiao P., Tian Y., Zhang W., Zhang S., Hou F., Bao Z.-W. (2025). Prognostic value of inflammatory markers for all-cause mortality in patients with acute myocardial infarction in the coronary care unit: A retrospective study based on MIMIC-IV database. Front. Cardiovasc. Med..

[10] Armstrong E.J., Morrow D.A., Sabatine M.S. (2006). Inflammatory biomarkers in acute coronary syndromes: Part II: Acute-phase reactants and biomarkers of endothelial cell activation. Circulation.

[11] Blake G.J., Ridker P.M. (2003). C-reactive protein and other inflammatory risk markers in acute coronary syndromes. J. Am. Coll. Cardiol..

[12] Larsen J.B., Hvas A.M. (2021). Fibrin clot properties in coronary artery disease: New determinants and prognostic markers. Pol. Arch. Intern. Med..

[13] Kietsiriroje N., Ariens R.A.S., Ajjan R.A. (2021). Fibrinolysis in Acute and Chronic Cardiovascular Disease. Semin. Thromb. Hemost..

[14] Sproston N.R., Ashworth J.J. (2018). Role of C-Reactive Protein at Sites of Inflammation and Infection. Front. Immunol..

[15] Yang P., Zhang Q.X., Huang Z.K. (2025). Predictive Value of Serum D-Dimer and Prothrombin Time for Adverse Events in Patients with Acute Myocardial Infarction. Clin. Lab..

[16] Chen R., Liu C., Zhou P., Tan Y., Sheng Z., Li J., Zhou J., Chen Y., Song L., Zhao H. (2021). Prognostic Value of D-dimer in patients with acute coronary syndrome treated by percutaneous coronary intervention: A retrospective cohort study. Thromb. J..

[17] Yu Z.Y., Chan P.K., Lin T.C., Hung Y., Yu F.H., Lin W.S., Cheng S.M., Lin W.Y. (2024). The Prognostic Value of D-Dimer in Patients with Acute Myocardial Infarction: A Retrospective Longitudinal Cohort Study in Taiwan. Acta Cardiol. Sin..

[18] Iqbal J., Wu H.X., Wu Y.X., Jiang H.L., Li L., Zhou X.Y., Bu Y.H., Zhou H.D. (2026). MetsObesity: A novel classification system for predicting 15-year cardiovascular risk in the UK Biobank population. J. Adv. Res..

[19] Biccire F.G., Farcomeni A., Gaudio C., Pignatelli P., Tanzilli G., Pastori D. (2021). D-dimer for risk stratification and antithrombotic treatment management in acute coronary syndrome patients: Asystematic review and metanalysis. Thromb. J..

[20] Huang D., Gao W., Wu R., Zhong X., Qian J., Ge J. (2020). D-dimer level predicts in-hospital adverse outcomes after primary PCI for ST-segment elevation myocardial infarction. Int. J. Cardiol..

[21] Jurado O.M., Duran J., Martinez A., Castellon J.M., Gutierrez M.A. (2009). Acute myocardial infarction in a man without coronary atheromatosis and antiphospholipid syndrome: Report of one case. Rev. Med. Chile.

[22] Gurlek A., Ozdol C., Pamir G., Dincer I., Tutkak H., Oral D. (2005). Association between anticardiolipin antibodies and recurrent cardiac events in patients with acute coronary syndrome. Int. Heart J..

[23] Abid L., Frikha F., Bahloul Z., Kammoun S. (2011). Acute myocardial infarction in young adults with antiphospholipid syndrome: Report of two cases and literature review. Pan Afr. Med. J..

[24] Barreno-Rocha S.G., Guzman-Silahua S., Rodriguez-Davila S.D., Gavilanez-Chavez G.E., Cardona-Munoz E.G., Riebeling-Navarro C., Rubio-Jurado B., Nava-Zavala A.H. (2022). Antiphospholipid Antibodies and Lipids in Hematological Malignancies. Int. J. Mol. Sci..

[25] Veres K., Lakos G., Kerenyi A., Szekanecz Z., Szegedi G., Shoenfeld Y., Soltesz P. (2004). Antiphospholipid antibodies in acute coronary syndrome. Lupus.

[26] Che J., Li G., Wang W., Li Q., Liu H., Chen K., Liu T. (2011). Serum autoantibodies against human oxidized low-density lipoproteins are inversely associated with severity of coronary stenotic lesions calculated by Gensini score. Cardiol. J..

[27] Santos A.O., Fonseca F.A., Fischer S.M., Monteiro C.M., Brandao S.A., Povoa R.M., Bombig M.T., Carvalho A.C., Monteiro A.M., Ramos E. (2009). High circulating autoantibodies against human oxidized low-density lipoprotein are related to stable and lower titers to unstable clinical situation. Clin. Chim. Acta.

[28] Millard R.W., Tranter M. (2014). Complementary, alternative, and putative nontroponin biomarkers of acute coronary syndrome: New resources for future risk assessment calculators. Rev. Esp. Cardiol. (Engl. Ed.).

[29] Ordóñez J., Genís A.B. (2015). Cien Años de Historia del Infarto de Miocardio Contada a Través de sus Biomarcadores.

[30] Stanojkovic A., Mrdovic I., Tosic I., Matic D., Savic L., Petrovic J., Cirkovic A., Milosevic A., Srdic M., Kostic N. (2025). Prognostic Value of the RISK-PCI Score in Patients with Non-ST-Segment Elevation Acute Myocardial Infarction. J. Clin. Med..

[31] Ginanjar E., Hustrini N.M., Mansjoer A., Al Hanif M.S. (2023). Factors Associated with 30-day Major Adverse Cardiovascular Event in Acute Coronary Syndrome Patients with Non-Dialysis Chronic Kidney Disease: A Retrospective Cohort Study. Acta Med. Indones..

[32] Gulati M., Levy P.D., Mukherjee D., Amsterdam E., Bhatt D.L., Birtcher K.K., Blankstein R., Boyd J., Bullock-Palmer R.P., Conejo T. (2021). 2021 AHA/ACC/ASE/CHEST/SAEM/SCCT/SCMR Guideline for the Evaluation and Diagnosis of Chest Pain: A Report of the American College of Cardiology/American Heart Association Joint Committee on Clinical Practice Guidelines. Circulation.

[33] Kim Y., Ahn Y., Cho M.C., Kim C.J., Kim Y.J., Jeong M.H. (2019). Current status of acute myocardial infarction in Korea. Korean J. Intern. Med..

[34] Doggen C.J., Zwerink M., Droste H.M., Brouwers P.J., van Houwelingen G.K., van Eenennaam F.L., Egberink R.E. (2016). Prehospital paths and hospital arrival time of patients with acute coronary syndrome or stroke, a prospective observational study. BMC Emerg. Med..

[35] Collinson P., Dakshi A., Khand A. (2023). Rapid diagnostic strategies using high sensitivity troponin assays: What is the evidence and how should they be implemented?. Ann. Clin. Biochem..

[36] Cediel G., Rueda F., Garcia C., Oliveras T., Labata C., Serra J., Nunez J., Bodi V., Ferrer M., Lupon J. (2017). Prognostic Value of New-Generation Troponins in ST-Segment-Elevation Myocardial Infarction in the Modern Era: The RUTI-STEMI Study. J. Am. Heart Assoc..

[37] Kozinski M., Krintus M., Kubica J., Sypniewska G. (2017). High-sensitivity cardiac troponin assays: From improved analytical performance to enhanced risk stratification. Crit. Rev. Clin. Lab. Sci..

[38] Lazar D.R., Lazar F.L., Homorodean C., Cainap C., Focsan M., Cainap S., Olinic D.M. (2022). High-Sensitivity Troponin: A Review on Characteristics, Assessment, and Clinical Implications. Dis. Markers.

[39] Torralba F., Navarro A., la Hoz J.C., Ortiz C., Botero A., Alarcon F., Isaza N., Isaza D. (2020). HEART, TIMI, and GRACE Scores for Prediction of 30-Day Major Adverse Cardiovascular Events in the Era of High-Sensitivity Troponin. Arq. Bras. Cardiol..

[40] Badimon L., Pena E., Arderiu G., Padro T., Slevin M., Vilahur G., Chiva-Blanch G. (2018). C-Reactive Protein in Atherothrombosis and Angiogenesis. Front. Immunol..

[41] Shibata N., Glass C.K. (2009). Regulation of macrophage function in inflammation and atherosclerosis. J. Lipid Res..

[42] Kaura A., Hartley A., Panoulas V., Glampson B., Shah A.S.V., Davies J., Mulla A., Woods K., Omigie J., Shah A.D. (2022). Mortality risk prediction of high-sensitivity C-reactive protein in suspected acute coronary syndrome: A cohort study. PLoS Med..

[43] Mani P., Puri R., Schwartz G.G., Nissen S.E., Shao M., Kastelein J.J.P., Menon V., Lincoff A.M., Nicholls S.J. (2019). Association of Initial and Serial C-Reactive Protein Levels With Adverse Cardiovascular Events and Death After Acute Coronary Syndrome: A Secondary Analysis of the VISTA-16 Trial. JAMA Cardiol..

[44] Yang S., Pan Y., Zheng W. (2025). Baseline High-Sensitivity C-Reactive Protein as a Predictor of Adverse Clinical Events in Patients with Coronary Artery Disease Undergoing Percutaneous Coronary Intervention: A Meta-Analysis. Cardiol. Rev..

[45] Iyer K.S., Dayal S. (2020). Modulators of platelet function in aging. Platelets.

[46] Kowalska M.A., Rauova L., Poncz M. (2010). Role of the platelet chemokine platelet factor 4 (PF4) in hemostasis and thrombosis. Thromb. Res..

[47] Brambilla M., Josefsson E.C., Ramstrom S., Di Minno A., Di Minno M.N.D., Gangatirkar P., Moujalled D., Becchetti A., Lordkipanidze M., Camera M. (2025). Biomarkers of in vivo platelet activation in coronary artery disease: A systematic review and meta-analysis: Communication from the SSC of the ISTH. J. Thromb. Haemost..

[48] Iseme R.A., McEvoy M., Kelly B., Agnew L., Walker F.R., Handley T., Oldmeadow C., Attia J., Boyle M. (2017). A role for autoantibodies in atherogenesis. Cardiovasc. Res..

[49] Antolín J., Almeida D., José Amérigo M., Cantabrana A., Roces A., Hayeck M. (2002). Anticuerpos anti-β2-glucoproteina I y lupus eritematoso sistemico. Med. Clín..

[50] Barbhaiya M., Zuily S., Naden R., Hendry A., Manneville F., Amigo M.C., Amoura Z., Andrade D., Andreoli L., Artim-Esen B. (2023). The 2023 ACR/EULAR Antiphospholipid Syndrome Classification Criteria. Arthritis Rheumatol..

[51] Greco T.P., Conti-Kelly A.M., Greco T., Doyle R., Matsuura E., Anthony J.R., Lopez L.R. (2009). Newer antiphospholipid antibodies predict adverse outcomes in patients with acute coronary syndrome. Am. J. Clin. Pathol..

[52] Association W.M. (2025). World Medical Association Declaration of Helsinki: Ethical Principles for Medical Research Involving Human Participants. JAMA.

